# Spatial distribution and cluster analysis of retail drug shop characteristics and antimalarial behaviors as reported by private medicine retailers in western Kenya: informing future interventions

**DOI:** 10.1186/s12942-016-0038-8

**Published:** 2016-02-19

**Authors:** Andria Rusk, Linda Highfield, J. Michael Wilkerson, Melissa Harrell, Andrew Obala, Benjamin Amick

**Affiliations:** The University of Texas School of Public Health, Pressler Dr, Houston, TX USA; Moi University School of Medicine, Nandi Rd, Eldoret, Kenya; Webuye Demographic Surveillance Site Scientific Steering Committee, Eldoret, Kenya; Department of Health Policy and Management, Florida International University, Robert Stempel College of Public Health and Social Work, Miami, FL USA; Institute for Work and Health, Toronto, Canada

**Keywords:** Cluster analysis, Antimalarial, Retail shops, Medicine outlets, Scan statistic, Geospatial

## Abstract

**Background:**

Efforts to improve malaria case management in sub-Saharan Africa have shifted focus to private antimalarial retailers to increase access to appropriate treatment. Demands to decrease intervention cost while increasing efficacy requires interventions tailored to geographic regions with demonstrated need. Cluster analysis presents an opportunity to meet this demand, but has not been applied to the retail sector or antimalarial retailer behaviors. This research conducted cluster analysis on medicine retailer behaviors in Kenya, to improve malaria case management and inform future interventions.

**Methods:**

Ninety-seven surveys were collected from medicine retailers working in the Webuye Health and Demographic Surveillance Site. Survey items included retailer training, education, antimalarial drug knowledge, recommending behavior, sales, and shop characteristics, and were analyzed using Kulldorff’s spatial scan statistic. The Bernoulli purely spatial model for binomial data was used, comparing cases to controls. Statistical significance of found clusters was tested with a likelihood ratio test, using the null hypothesis of no clustering, and a *p* value based on 999 Monte Carlo simulations. The null hypothesis was rejected with *p* values of 0.05 or less.

**Results:**

A statistically significant cluster of fewer than expected pharmacy-trained retailers was found (RR = .09, *p* = .001) when compared to the expected random distribution. Drug recommending behavior also yielded a statistically significant cluster, with fewer than expected retailers recommending the correct antimalarial medication to adults (RR = .018, *p* = .01), and fewer than expected shops selling that medication more often than outdated antimalarials when compared to random distribution (RR = 0.23, *p* = .007). All three of these clusters were co-located, overlapping in the northwest of the study area.

**Conclusion:**

Spatial clustering was found in the data. A concerning amount of correlation was found in one specific region in the study area where multiple behaviors converged in space, highlighting a prime target for interventions. These results also demonstrate the utility of applying geospatial methods in the study of medicine retailer behaviors, making the case for expanding this approach to other regions.

## Background

Seeking initial treatment for febrile illness from retail drug outlets is a common practice in many regions where malaria is endemic [1–6]. While efforts are being made to ensure availability of effective firstline malaria treatments in drug outlets, several studies have found private medicine retailers continue recommending, prescribing, and dispensing outdated antimalarials [7–11]. Consequently, many programs have been implemented to increase medicine retailer knowledge and modify their practices [12, 13]. The programs come at a high cost, and could be more effective when efforts consider local environments [14] and are adapted to the local context [15].

Geospatial analysis techniques, specifically cluster analysis, can help improve effectiveness and adaptability by identifying specific spatial areas where intervention is most needed, and thus helping target financial resources. While these methods have been used frequently to detect malaria disease clusters [16–20], they have been employed less frequently to study malaria-related behaviors [21, 22], and have not yet been expanded to explore private medicine retailer behaviors.

The Affordable Medicines Facility—Malaria (AMFm) program, which subsidized costly artemisinin-based combination therapies (ACTs) to encourage their prescription and dispensation over cheaper but less effective therapies for treating malaria, was recently transitioned into the Global Fund’s existing core grant processes [23]. A change from subsidies to a co-payment mechanism will require countries that had previously received subsidies through the AMFm program that reduced the cost of ACTs, to now choose how much of their country’s budget will be re-allocated from existing priorities to pick up the cost of subsidizing these medications [24]. It is expected that costs of ACTs will rise, and where cost plays a role in which drugs are dispensed or purchased, will reduce the use of ACTs and increase the use of monotherapies or other less-effective medicines [24]. This, in combination with the $109 million spent in Kenya in 2009 on malaria treatment and prevention in children alone [25], has resulted in calls for innovative approaches to improve private retailer services without posing significant financial challenges to local governments [26].

It is now more important than ever to be able to target interventions aimed at modifying medicine retailer behavior toward recommending and dispensing effective medications (and not recommending or dispensing ineffective medications). Discovering any spatial clustering of private medicine retailer behaviors and other predictors of appropriate dispensing behavior will provide critical intelligence to target interventions, increase intervention effectiveness, and reduce costs. The study objectives are to describe spatial clustering of specific retail drug shop characteristics and medicine retailer knowledge and behaviors that are related to appropriate malaria case management.

## Methods

### Study area and sample

**Fig. 1:**
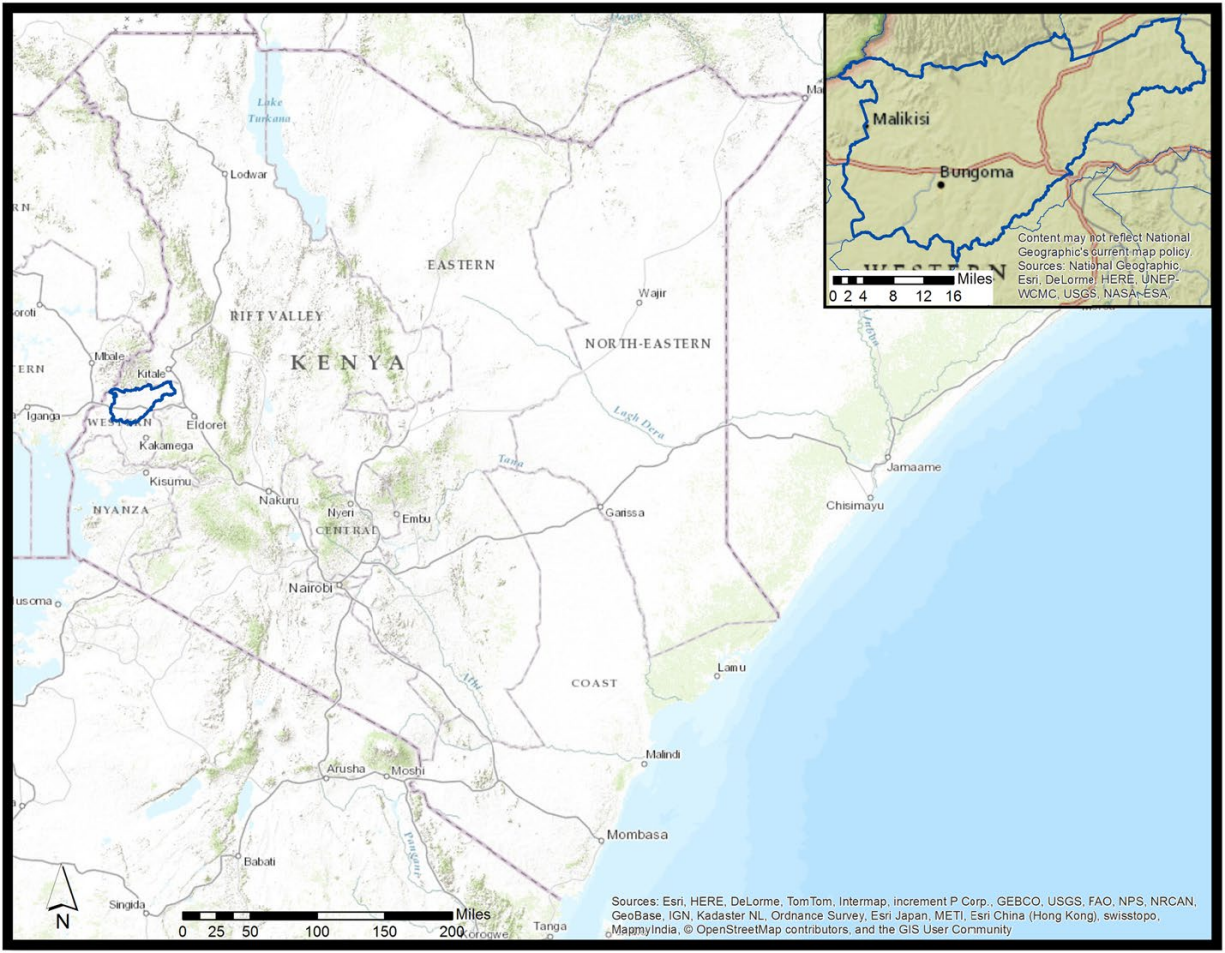
Map of Kenya, with the Bungoma district containing the study area inset

The data for the present study are from the Webuye Health and Demographic Surveillance Site (WHDSS) in the Bungoma district of Kenya’s Western Province [27]. The study area is home to roughly 80,000 residents [28], and has a total area of 130 km [2] (50.2mi^2^) [29]. The site sits at an elevation of 1523 m (4997ft) with a range of 1477–1733 m [30], and lies at 0.617° latitude and 34.767° longitude [28]. A map of Kenya, with the Bungoma district that contains the study region set as an inset, is available in Fig. 1. The region’s primary economy is subsistence farming and a single local sugar-processing plant. This area is endemic for malaria, and suffers from a particularly high burden of malarial disease. A 1998 study of western Kenya found *P. falciparum* parasites in 44 % of asymptomatic children during the dry season, and 55.4 % of children in the wet season [31]. A detailed description of the WHDSS has been published previously [29].

The WHDSS area is largely rural with one small peri-urban center located just beyond the WHDSS boundary, and includes one district hospital, one faith-based hospital, one health center, two medicine dispensaries, and multiple businesses serving the private health sector. The retail sector includes privately owned pharmacies, traditional healers, herbalists, chemists, agrovets that also carry human medicines, and drug outlets.

**Fig. 2:**
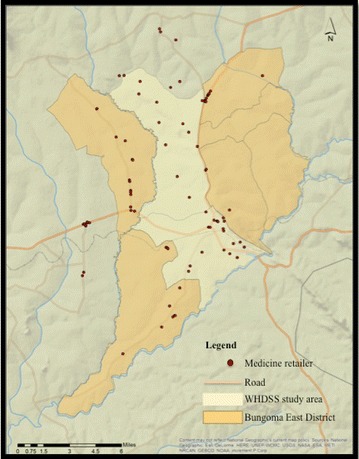
Detailed map of the study area with shop locations. Adapted from Smith et al. 2011 [50]

These locations, referred to here as medicine retailers, make up the study population and include all outlets that are located up to or within 5 km of the WHDSS border with the exception of the river-bound north-eastern border, to include the retailers that are accessible to those living within the WHDSS. Locations that were included were privately owned and carried any antimalarial medication. Exclusion criteria included those retailers that sold only general goods, were public health facilities, or refused to participate in the survey. A map of the WHDSS, retail outlet locations, and major roadways can be seen in Fig. 2.

### Data collection

Each research team member was assigned to cover a sub-section of the study area on motorbike. Team members began in the market center of their area and identified all medicine retailers in that area. The name and GPS coordinates of each location were recorded. In each retail drug shop, retailers were asked to identify any other places to buy medicines nearby. This process of drug shop identification continued until no new locations were identified. Team members then visited each identified location to administer a survey. The first respondent at each shop completed the survey. A Garmin E-trex handheld GPS unit was used to map all the major roads, town centers, and retail drug shop locations in the study area.

### Measures

Meetings were held with prominent members of the community, including chiefs, assistant chiefs, and village elders to review survey objectives and seek their approval prior to study commencement. These leaders circulated study information to their communities. In addition, all participants in the study gave verbal consent before participating in the survey.

The survey contained eighty items, all of which were pretested outside the study area. The survey items included for analysis collected medicine retailer characteristics, retailer behaviors, and retail drug shop characteristics that related to malaria case management. These variables were selected for exploration based on findings in the literature that show these variables as significant predictors of access to appropriate care for malaria [12, 14].

#### Medicine retailer characteristic measures

Retailer characteristics included health qualification, type of health qualification, education level, and antimalarial drug knowledge. The health qualification data were captured with the question, “Do you have a health-related qualification?” Recorded responses were “yes” (coding = 1), and “no” (coding = 0).

The type of health qualification variable was comprised of a two-part question. Only those who answered in the affirmative to the first question, “Do you have a health-related qualification?” were then asked the second question, “What is your type of qualification?” Open-ended responses were categorized into “Pharmacist,” “Pharmacy technician,” “Pharmacy assistant,” “Medical Doctor,” “Nurse/Midwife,” “Clinical Officer,” and “Other.” For the purposes of this research, responses were collapsed into two categories, “Pharmacy-trained,” which included all open-ended responses referencing any training in pharmacy (coding = 1), and “Nurse/Midwifery-trained,” which included all open-ended responses referencing any training in nursing or midwifery (coding = 0).

The education variable was an open-ended question: “What is your education level?” Responses were recorded, and categorized into “None,” “Some primary,” “completed primary,” “complete secondary,” or “some or completed above secondary.” For the purposes of this research, the first four categories were collapsed into one group; “Completed secondary or less” (coding = 0). The last category remained the same: “Some or completed above secondary” (coding = 1). This dichotomization was employed because the health-related training needed to operate a retail drug shop required at least a secondary school education [32].

The last respondent characteristic variable on antimalarial drug knowledge was based on the open-ended question, “What is the name of the malaria medication recommended by the Ministry of Health to treat uncomplicated malaria?” Responses were categorized into “ACT” or “Not ACT” according to the Ministry of Health (MOH) guidelines of using ACTs as the firstline therapy for treating uncomplicated malaria [33]. Because this question was open-ended, any response that met the MOH recommendations was included in the “ACT” category (coding = 1), including responses referring to Coartem, a brand name ACT medication, and Artemether Lumefantrine, or AL, referring to the generic name for Coartem. All other responses that did not reference an ACT medication were classified into the “Not ACT” category (coding = 0).

#### Medicine retailer behavior measures

The medicine retailer behaviors included in the analysis were recommending the correct antimalarial drug to a child under the age of five, and recommending the correct antimalarial drug to adults. These were both based on the open-ended questions “Which malaria medicine do you recommend most for children under 5 years of age” and “Which malaria medicine do you recommend most for adults,” respectively. Responses were classified in the same manner as the above question on antimalarial drug knowledge, into “ACT” and “Not ACT” categories, using the same coding.

#### Retail drug shop characteristic measures

The last variables captured shop characteristics, including whether a shop sold ACTs more than any other antimalarial, and whether the shop offered diagnostic services. The first of these was captured with an open-ended question, “Even if it is currently out of stock, which malaria medicine is most sold in your shop?” These responses were classified using the same approach as the above question on antimalarial drug knowledge, into “ACT” and “Not ACT” categories, using the same coding.

Offering diagnostic services was determined with two open-ended questions: “Do you ever provide microscopic testing on the premises” and “Do you ever sell Rapid Diagnostic Testing kits?” If a response was affirmative to either question, that response was classified as “Offers diagnostic testing” (coding = 1). If a response was negative to both questions, that response was classified as “Does not offer diagnostic testing” (coding = 0).

### Data entry and analysis

Microsoft Access was used to record all data that was collected from the survey, which were double entered and confirmed. Discrepancies were resolved by consulting the original hard copy survey forms. Variables from the survey included in this study were coded and imported into Stata v11 [34]. After coding, variables were exported from Stata and imported into Kulldorff’s SaTScan program version 9.3.1 [35, 36].

Kulldorff’s spatial scan statistic was used due to its wide application in public health [37], and malaria research [16–18, 20]. The Bernoulli purely spatial model for binomial data was used, which compares cases to controls. Cases are defined as those responses coded as a “1.” Controls are those responses coded as a “0” [38, 39]. The scan statistic allows for the detection of clustering of cases within a circular window that differs significantly from the expected random distribution, yielding the window (or windows) with the highest value. SaTScan tests the hypothesis of detecting statistically significant clusters against the null hypothesis of no significant clusters.

SaTScan tests this hypothesis by creating a series of circular windows over the study area, evaluating each one as a possible cluster. The window with the highest likelihood of being a cluster is assigned a *p* value adjusted for multiple testing [40]. The spatial scan is set to detect higher rates of the variable of interest, called “hot spots”, where there are more cases found within the cluster than would be expected, as well as lower rates, called “cold spots,” where there are fewer cases found than would be expected, determined using an alpha of 0.05. Statistical significance of clusters is tested with a likelihood ratio test, using the null hypothesis of no clustering (random distribution), and a *p* value based on 999 Monte Carlo simulations [40]. The null hypothesis was rejected with *p* values of 0.05 or less.

Clustering was tested for all variables at both high and low rates. Only the low rates were reported here, because it is the low rates of key behaviors that are most appropriate for informing interventions. Also, since all included variables were binary, the results for low rate clusters were the inverse of the results for high rates of that same variable, so reporting both was deemed unnecessary.

The default setting of SaTScan for the maximum window size is no more than 50 % of the study population. This parameter can be modified by the user, yet no guidance is given within the program manual recommending any criteria to be used to inform this selection. The need for guiding principles to help choose an appropriate maximum window size has been the discussion of past methodological papers [37, 41]. Since this area is heterogeneous on a large scale, with varying population density around market centers and peri-urban regions, but homogenous on a smaller scale when focused on a specific sub-region, it is important that detection efforts are flexible, and are able to detect effects on multiple scales.

For the purposes of this research, variable clusters were run on maximum window sizes from 50 to 15 %, using increments of 5 %. Stability of the significant cluster was determined by comparing the results of each run, and was defined by the primary cluster maintaining size and space over repeated scans [37]. Only the primary significant clusters were reported as the software did not identify any statistically significant secondary clusters that did not overlap with primary clusters [42].

With one exception, all clustering of individual-level variables were reported at the 25 % maximum window size. This scan parameter was selected for the purposes of informing interventions, and because the primary SaTScan cluster for these variables did not change size or space for any intervals of 5 % between 50 and 15 % maximum window size. Additionally, the stability of these clusters lends reliability to the selection of a scan at any interval [37]. The one exception is with low rates of nursing or midwifery-trained retailers compared to pharmacy-trained retailers. This variable was reported at the 20 % maximum window size.

All clusters of shop-level variables, whether the sold ACTs more than any other antimalarial and whether they ever offered diagnostic testing, were reported at the 50 % maximum window size. This size was chosen because these two variables had low numbers of cases in their clusters. This maximum window size was selected both to retain power, and to best inform interventions by capturing more cases.

For each variable analyzed, the location of the statistically significant cluster was reported, along with the observed number of cases within the cluster, and the expected number of cases within that cluster. Also reported were the relative risks for each variable cluster, where the numerator was the risk of being a case within the cluster, and the denominator was the risk of being a case outside the cluster. It was calculated as the observed cases divided by the expected cases within the cluster divided by the observed cases divided by the expected cases outside the cluster [43]. Where there were zero cases within a cluster, the relative risk will always be zero. These were also reported.

### Ethics

The data used in this study, and the research effort that led to its collection, was reviewed and approved by the Moi University Institutional Research and Ethics Committee (Reference# IREC/2008/05). This research was reviewed, approved, and deemed exempt from The University of Texas Health Science Center at Houston Committee for the Protection of Human Subjects (Reference# HSC-SPH-14-1035). All medicine retailers included in the study gave verbal consent prior to participating in the survey.

## Results

Medicine retailers from 97 distinct outlets participated in the survey. Four observations were excluded from the analysis because GIS coordinates were not recorded for those surveys. Two additional observations were dropped from the analysis because the latitude and longitude were recorded incorrectly (recording points over the Indian Ocean), and because the intended coordinates could not be known, they were excluded. Of the 91 medicine retailers included in the analysis, 70 were women, 12 had no health-related training, 31 had no education beyond secondary school, and roughly half were the shop owner. Medicine retailer characteristics are summarized in Table 1. Retail drug shops had anywhere from one to six employees, with a mean of 1.5. Nine percent of shops had ever offered microscopic testing, and only 2 % had ever offered rapid diagnostic testing. Retail drug shop characteristics are summarized in Table 2.

**Table 1:** Medicine retailer characteristics

	N = 91	%
Age		
Under 30	n = 34	37
30–50	n = 49	54
Over 50	n = 7	8
Missing	n = 1	1
Female ratio	n = 70	77
Education		
Secondary school or less	n = 31	34
More than secondary school	n = 60	66
Training		
Pharmacy trained	n = 37	41
Nursing/midwifery trained	n = 42	46
Untrained	n = 12	13
Years worked in the shop		
1 year or less	n = 32	35
1–5 years	n = 38	42
5–10 years	n = 14	15
10+ years	n = 7	8
Facility owner		
Yes	n = 46	51
No	n = 45	49

**Table 2:** Retail drug shop characteristics

	N = 91	%
Number of staff—Mean (min–max)	1.49 (1–6)	
Number who dispense—Mean (min–max)	1.22 (1–2)	
Ever outage of antimalarials		
Yes	n = 40	44
No	n = 51	56
Ever offered microscopic testing		
Yes	n = 8	9
No	n = 83	91
Ever offered rapid diagnostic testing		
Yes	n = 2	2
No	n = 89	98

### Distribution of spatial clustering

Of the nine variables included in the analysis, seven of them yielded statistically significant clusters. The two variables that did not show statistically significant clustering were having any health-related training and recommending appropriate malaria treatment to children. The cluster analysis of all variables are summarized in Table 3.

**Table 3:** Results of the cluster analysis

Retail drug shop characteristics	High rate or low rate	No. of cases in cluster	No. of expected cases	No. of controls in cluster	Log likelihood ratio	Relative risk	*p* value
Sells ACTs more than any other antimalarial	Low	4	12.54	28	8.24	0.23	.007*
Offers diagnostic testing (MST or RDT)	Low	0	4.45	45	6.62	0	.014*
Medicine retailer characteristics							
Has more than a secondary school education	Low	0	8.57	13	16.24	0	<.001*
Has health-related training	Low	0	1.74	2	4.21	0	.352
Is trained in pharmacy, rather than nursing/midwifery	Low	1	8.9	22	10.3	0.09	.001*
Is trained in nursing/midwifery rather than pharmacy	Low	0	4.25	8	6.58	2.45	.053*
Did identify ACTs as the MOH-recommended firstline antimalarial for non-complicated malaria	Low	0	4.4	7	7.46	0	.025*
Medicine retailer behaviors							
Would recommend appropriate malaria treatment for children under 5	Low	2	5.86	11	2.98	0.31	.877
Would recommend appropriate malaria treatment for adults	Low	2	9.34	15	8.5	0.18	.010*

* Statistical significance at *p* < 0.05

#### Medicine retailer characteristics

**Fig. 3:**
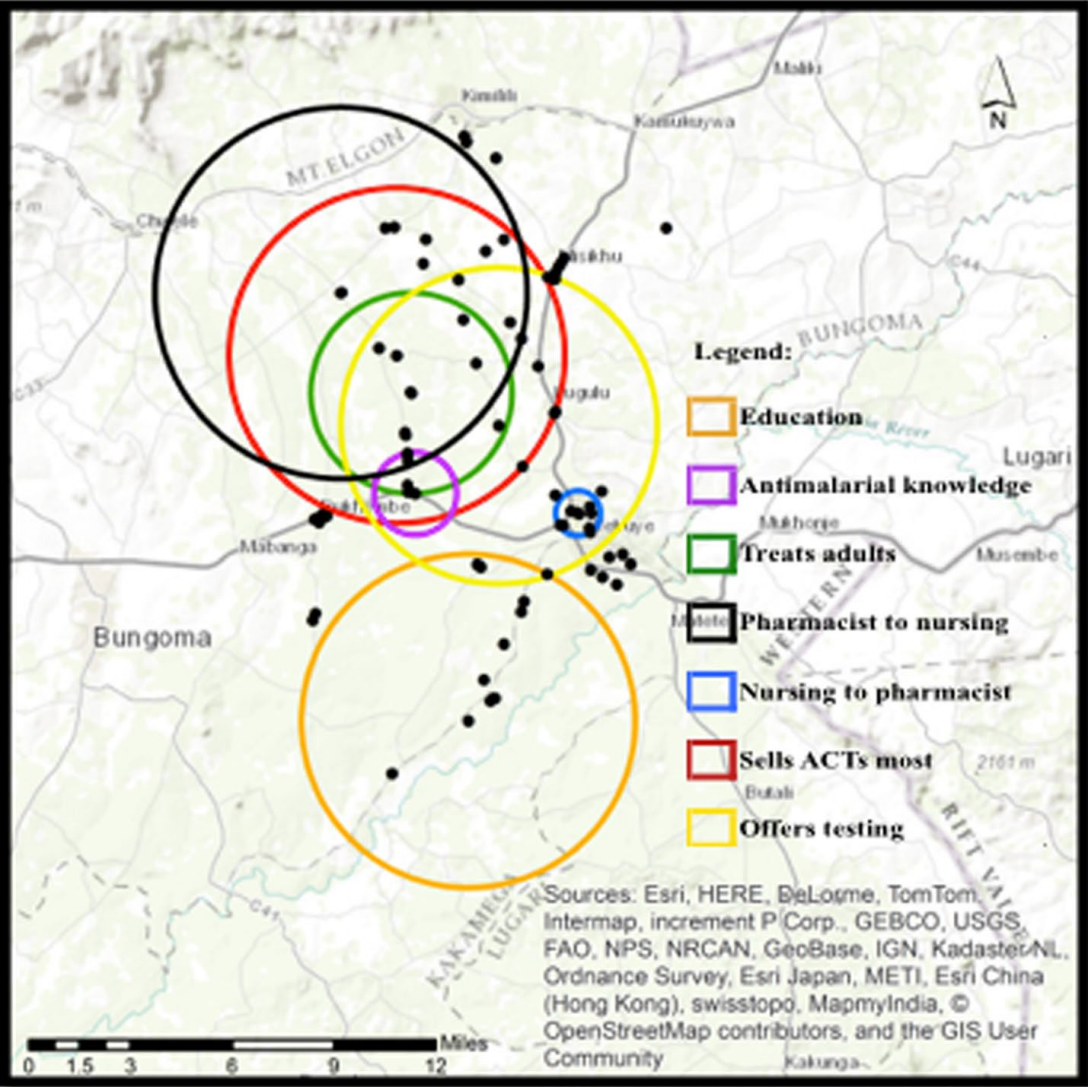
Geographic position of statistically significant clusters for low rates of included variables

Statistically significant clusters were found for the following medicine retailer characteristics: education, type of health training (whether in pharmacy or nursing/midwifery), and antimalarial drug knowledge (or, correctly identifying ACTs as the recommended antimalarial therapy). Only one of these four, education, was clustered in the south of the study area. The remaining three were clustered in the northwest of the study area. Also, only one of the four, having nursing/midwifery health training, was clustered in the peri-urban center of Webuye Town. The remaining variables clustered in non-urban areas. Please see Fig. 3 for a geographic representation of the location for each statistically significant cluster.

##### Medicine retailer education

The education cluster compared those retailers who reported an education beyond secondary school to those who stopped at secondary school. The statistically significant cluster contained zero cases, but had an expected 8.57 cases (RR = 0.00, *p* < .001). This region of the study area had far fewer retailers with above a secondary education than was expected given the distribution of education across the rest of the study area.

##### Medicine retailer health-related training

The pharmacy-trained cluster compared those with training in pharmacy to those with training in nursing or midwifery. This “cold spot” cluster was located in the northwest of the study area, and was also off the major roadways. The statistically significant cluster contained 1 case (or retailer trained in pharmacy), to an expected 8.9 cases (RR = .09, *p* = .001). Working in a shop within that cluster reduced the likelihood of being trained in pharmacy by 91 %, also indicating a higher-than expected concentration of nursing/midwifery-trained retailers.

The nursing/midwifery-trained cluster compared those with training in nursing/midwifery to those with training in pharmacy. The statistically significant cluster had zero cases, to an expected 4.25 (RR = 0.0, *p* = .053), indicating there were fewer than expected nursing-trained retailers working in Webuye Town. This was the only variable that clustered in the peri-urban area.

This was also the only retailer-level variable not reported at the 25 % maximum window size. These results are for the 20 % maximum window size. When windows of larger sizes were scanned, the results lost statistical significance.

##### Antimalarial drug knowledge

The malaria drug knowledge cluster, which found lower rates than expected of medicine retailers who correctly identified the MOH-recommended antimalarial for uncomplicated malaria compared to those who could, contained zero cases compared to an expected 4.4 cases based on simulations (RR = 0.00, *p* = .025). This cluster was also in the northwest of the study area, and overlaps considerably with the cluster of nursing/midwifery-trained retailers.

#### Medicine retailer behaviors

##### Recommending correct treatment to adults

The only retailer behavior variable that had a statistically significant cluster was recommending the correct antimalarial treatment to adults. This cluster found lower than expected rates of retailers who would recommend correct antimalarial treatment to adults, with only 2 cases inside the cluster out of an expected 9.34. Working in a shop inside this cluster increased your “risk” of recommending inappropriate treatment to adults by 82 % (RR = 0.18, *p* = .01). This cluster was located in the northwest of the study area, overlapping with low rates of knowing the correct antimalarial therapy, and low rates of having training in pharmacy.

##### Retail drug shop characteristics

Cluster analyses also revealed statistically significant clusters for low rates in both shop-level variables: whether a shop offered diagnostic testing, and whether a shop sold ACTs more than any other antimalarial. As described, both of these clusters are reported at the 50 % maximum window size to retain power and intervention utility.

##### Offered diagnostic testing

Lower than expected rates of offering diagnostic testing were found to be geospatially clustered in the study area. This cluster contained zero observed cases, but 4.45 expected cases (RR = 0.00, *p* = .014). This was the only cluster that included both peri-urban and rural shop locations.

##### Sold ACTs more than other antimalarials

The second shop level variable of selling ACTs more than other antimalarials was found to cluster across space. This cluster contained four observed cases, but 12.54 expected cases (RR = 0.23, *p* = .007). The “risk” of selling a non-recommended antimalarial more than any other increased by 77 % within this cluster. The geographic location of this cluster reveals considerable overlap with having a nursing/midwifery-training, and with not recommending appropriate malaria treatment to adults.

**Fig. 4:**
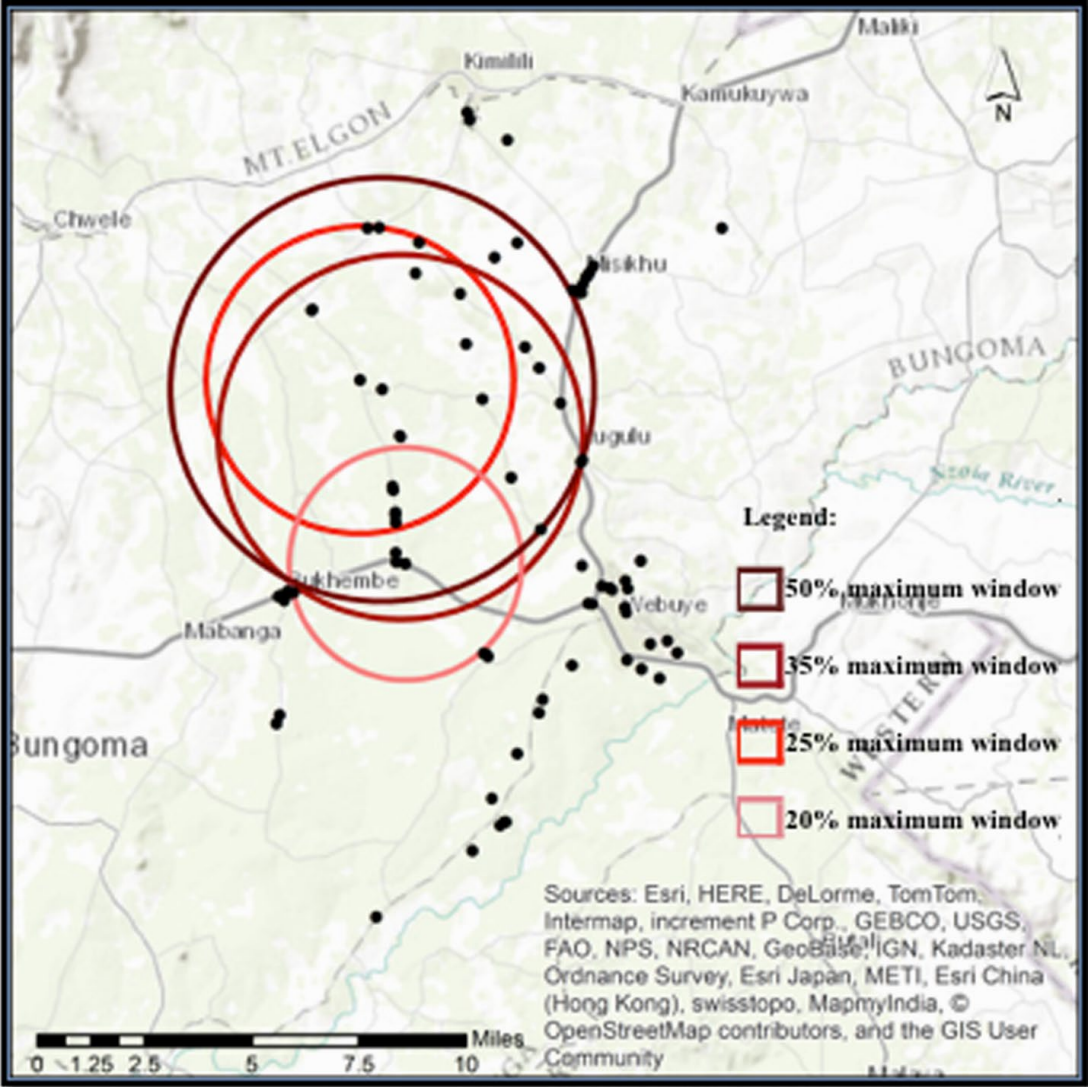
Cluster analysis results of varying intervals of maximum window sizes for single variable: Shop selling ACTs more than any other antimalarial

Even though this cluster was also reported at the 50 % maximum window size, selection of the appropriate scan size proved difficult in this case. This variable had two statistically significant clusters at all intervals between 50 and 30 %, but the primary cluster changed size and location as the window size was reduced. Once the 20 % scan was reached, the two clusters collapsed into one, and this single cluster remained statistically significant down to a window size of 15 %. Figure 4 depicts the movement of the primary cluster across each scan window, and its eventual joining with the secondary cluster at the 20 % window size.

As the primary cluster moved across space, the *p* values did change slightly with each change in geographic location of the cluster. However, statistical significance was retained across all clusters. The reported relative risk and *p* value at the 50 % maximum window size remain unchanged at 45 %. At 35 %, the relative risk drops to 0.22 and the *p* value increases to 0.018. Table 4 summarizes the results of the cluster analysis for this variable at each maximum window size, and includes comparisons of the relative risk, *p* value, and changes to expected and observed numbers of cases within each cluster.

**Table 4:** Results of cluster analysis at 5 % maximum window size intervals

Maximum window	Size significant cluster order	Relative risk	Log likelihood ratio	*p* value	No. of cases in cluster	No. of expected cases	No. of controls in cluster	Radius of cluster
*50*	*Primary*	*0.32*	*8.24*	*0.007*	*4*	*12.54*	*32*	*7.97*
*50*	*Secondary*	*0*	*6.85*	*0.038*	*0*	*4.85*	*12*	*4.37*
45	Primary	0.23	8.24	0.007	4	12.54	32	7.97
45	Secondary	0	6.85	0.038	0	4.85	12	4.37
40	Primary	0.23	8.24	0.006	4	12.54	32	7.97
40	Secondary	0	6.85	0.036	0	4.85	12	4.37
*35*	*Primary*	*0.22*	*7.16*	*0.018*	*3*	*10.51*	*27*	*6.86*
*35*	*Second*	*0*	*6.85*	*0.033*	*0*	*4.85*	*12*	*4.37*
30	Primary	0.22	7.16	0.017	3	10.51	27	6.86
30	Secondary	0	6.85	0.031	0	4.85	12	4.37
*25*	*Primary*	*0.11*	*6.99*	*0.018*	*1*	*7.28*	*19*	*5.78*
*25*	*Secondary*	*0*	*6.85*	*0.031*	*0*	*4.85*	*12*	*4.37*
*20* ^*a*^	*Primary*	*0*	*6.85*	*0.02*	*0*	*4.85*	*12*	*4.37*
15	Primary	0	6.85	0.02	0	4.85	12	4.37

Italicized areas are clusters shown in Fig. 2

^a^The primary cluster at 20 % is the same size and location of the secondary cluster for 50–25 %

## Discussion

The results of these analyses identified statistically significant clusters of key medicine retailer characteristics, behaviors, and retail drug shop characteristics that have been found to be predictive of antimalarial dispensing behaviors and access to appropriate malaria case management in past research [12, 14]. The results confirm the presence of spatial heterogeneity in these data. These findings can be used to inform interventions aimed at improving medicine retailer behaviors and malaria case management by enabling geographic targeting of a specific behavior requiring modification, rather than delivering multi-component interventions across the entire study area.

There was a statistically significant cluster of medicine retailers without a post-secondary education working in shops in the south of the study area, in the sub-locations of Makala and Milo. Agencies and others designing educational interventions for this region should consider targeting their efforts in these geographic areas, as they appear to contain most of the educational gap in this population. A surprising finding in regards to the education attained by medicine retailers is that there is no overlap between this and the geographic clusters of other variables. It is possible that education is not strongly associated with the included antimalarial behaviors, or that these relationships are not geospatial. Past research in this study area [7] did not find education to be a statistically significant predictor of antimalarial drug knowledge. However, for other intervention goals where education is a key predictor, those intervention efforts now have a clear geographic place to target their efforts.

While education was found to cluster in isolation from the other variables, an unexpected measure of correspondence was discovered in the remaining variables. Low rates of pharmacy-trained retailers overlapped with 100 % of the shop locations in the cluster of low rates of selling ACTs more than any other antimalarial. Also overlapping with these two clusters are the clusters of low rates of recommending the correct antimalarial treatment to adults, and low rates of knowing the correct antimalarial drug. These four variables have statistically significant clusters that all overlap with one another. This finding provides valuable information to interventionists because it explicates what behaviors should be targeted, and delivers a precise location where those programs are the most needed and would be of the most use.

With the concordance between the geospatial clustering of these variables, it is even more meaningful that the statistically significant cluster of low rates of nursing/midwifery-trained staff is entirely outside the region, and geospatial centered over the peri-urban center of Webuye Town. Here, lower than expected rates of nursing/midwifery-trained staff (which corresponds to higher than expected rates of pharmacy-trained staff) are not spatially co-located with the low rates of any other behaviors. While past research found pharmacy-trained staff to be more likely to know the correct antimalarial medication than untrained staff [7], it is not known if they are also more likely to have this knowledge, and to dispense accordingly, when compared to nursing or midwifery-trained staff. This is an issue that may have considerable implication on medicine retailer training programs and retail drug shop regulations, and requires further research.

Most clusters examined in the analyses are geographically centered away from major roadways. Clusters of low rates of education, appropriate treatment of adults, pharmacy-trained staff, and selling ACTs are positioned off major roads, and do not include shops situated on local highways or primary transportation routes. This posits that knowledge and training might have reached shops located on roadways better than shops in the interior, and that interventionists may need to apply more effort into infiltrating rural areas.

An urban/rural disparity is also evident in the results, with most clusters centered in the rural regions. The rural area of this study region had lower rates of retailers with pharmacy training, with a post-secondary school education, retailers who knew the MOH-recommended antimalarial, who would recommend that drug to adult patients, and who sold that drug more than other less-effective antimalarials. The urban part of the study region had higher rates of pharmacy-trained staff. This urban/rural variation is consistent with results from several other studies [10, 44–46], which found disparities in access to antimalarials and behavior of shop retailers between urban and rural shop locations.

A systematic review conducted by Wafula and colleagues [10] found that shops in urban locations are more likely to administer correct treatments, more likely to offer better services, and more likely to administer appropriate treatment than rural shops. Several other studies in sub-Saharan Africa have found that medicine retailers working in rural shop locations were more likely to dispense and stock medications according to customer demands rather than guidelines, were more likely to charge less for those medications, and were more likely to work in a drug shop rather than a pharmacy [47–49]. Urban locations were more likely to stock prescription-only drugs, were more likely to have antimalarial drugs on-hand, and were more likely to be co-located with a higher concentration of other healthcare facilities.

While the findings of this study affirm those of past research that urban/rural variation exists in medicine retailer behaviors and characteristics, they go beyond general rural and urban differences. They identify precisely where in the study area additional training is needed, and on specifically which behaviors. The urban/rural difference in these data is better contextualized by the spatially distributed clusters of characteristics and behaviors afforded by geospatial analysis.

Geospatial methods have been successfully employed in past research to identify geographic targets for interventions [40, 47–49], providing investigators with helpful information regarding where their interventions are most needed. While these activities are clear examples of the utility spatial analysis can have for intervention development, their use in the developing world, arguably where they could be most beneficial, is limited. These findings, and the foundation of applying geospatial methods to intervention development, encourage the expansion of this methodology into new fields. Additionally, the availability of the software used in this research, Kulldorff’s SaTScan program, is available for use without license cost, increases access to cluster analysis and expands its utility.

The results of this study provide insight that helps complete a more complete picture of medicine retailer behaviors related to appropriate malaria care, enabling efficient spatial targeting of training and intervention efforts aimed at increasing access to appropriate antimalarial medication and improving malaria case management. Knowing the geographic location of these “hot-spots” is critical to reducing the burden of malaria in this community. Given the new policy environment affecting available funds to support antimalarial access in the retail sector in Kenya, any opportunities to conserve funds through increased intervention efficiency are welcome discoveries.

## Conclusion

Malaria control and intervention efforts are costly, with the people of Kenya spending $109 million by the household and the health sector, on treating and preventing malaria in children alone [25]. Now more than ever, finding ways to make the most of money spent on interventions is critical. Using geospatial methods, such as cluster analysis, can provide valuable information to intervention developers, helping them target their programs, cover smaller areas, use fewer resources, and address key behaviors. Before embarking on costly programs, researchers should consider the utility of geospatial analytical techniques to help focus their efforts.

The building of geospatial databases can help facilitate future geospatial analysis studies by providing coordinates for key locations and variables of interest. Kenya’s demographic surveillance system undertakes a growing number of regularly administered surveys [29] that incorporate GIS data. The data collection efforts within the WHDSS serve as a role model to other regions looking to expand their own databases and opportunities for geospatial research.

Considering the exploratory nature of this study and the small geographic size of the study area, additional geospatial studies are needed, preferably with larger data sets, to investigate opportunities to incorporate geospatial relationships into intervention planning. Comparison studies should be made with those interventions that incorporate geospatial feedback for targeting, and those that do not.

The findings of this study provide insight into the true picture of medicine retailer behaviors in identifying specific areas where these behaviors need to be modified to best assure appropriate malaria case management. These results are actionable, and should be considered prior to the implementation of programs directed at these behaviors. While there has yet to be discovered a “silver bullet” to eradicate malaria, the employ of geospatial techniques may well provide a target.
